# Preclinical Development of a Novel Class of CXCR4 Antagonist Impairing Solid Tumors Growth and Metastases

**DOI:** 10.1371/journal.pone.0074548

**Published:** 2013-09-13

**Authors:** Luigi Portella, Rosamaria Vitale, Stefania De Luca, Crescenzo D’Alterio, Caterina Ieranò, Maria Napolitano, Anna Riccio, Maria Neve Polimeno, Luca Monfregola, Antonio Barbieri, Antonio Luciano, Andrea Ciarmiello, Claudio Arra, Giuseppe Castello, Pietro Amodeo, Stefania Scala

**Affiliations:** 1 Department of Oncological Immunology, ISTITUTO NAZIONALE PER LO STUDIO E LA CURA DEI TUMORI “Fondazione Giovanni Pascale”-IRCCS-ITALIA, Naples, Italy; 2 Animal Facility, ISTITUTO NAZIONALE PER LO STUDIO E LA CURA DEI TUMORI “Fondazione Giovanni Pascale”-IRCCS-ITALIA, Naples, Italy; 3 ICB-CNR, CNR, Pozzuoli, Italy; 4 IBB-CNR, CNR, Naples, Italy; 5 CROM, Mercogliano (AV), Italy; 6 Azienda Sanitaria Locale n 5 “Spezzino”, La Spezia, Italy; National Institutes of Health, United States of America

## Abstract

The CXCR4/CXCL12 axis plays a role in cancer metastases, stem cell mobilization and chemosensitization. Proof of concept for efficient CXCR4 inhibition has been demonstrated in stem cell mobilization prior to autologous transplantation in hematological malignancies. Nevertheless CXCR4 inhibitors suitable for prolonged use as required for anticancer therapy are not available. To develop new CXCR4 antagonists a rational, ligand-based approach was taken, distinct from the more commonly used development strategy. A three amino acid motif (Ar-Ar-X) in CXCL12, also found in the reverse orientation (X-Ar-Ar) in the vMIP-II inhibitory chemokine formed the core of nineteen cyclic peptides evaluated for inhibition of CXCR4-dependent migration, binding, P-ERK1/2-induction and calcium efflux. Peptides R, S and I were chosen for evaluation in *in vivo* models of lung metastases (B16-CXCR4 and KTM2 murine osteosarcoma cells) and growth of a renal cells xenograft. Peptides R, S, and T significantly reduced the association of the 12G5-CXCR4 antibody to the receptor and inhibited CXCL12-induced calcium efflux. The four peptides efficiently inhibited CXCL12-dependent migration at concentrations as low as 10 nM and delayed CXCL12-mediated wound healing in PES43 human melanoma cells. Intraperitoneal treatment with peptides R, I or S drastically reduced the number of B16-CXCR4-derived lung metastases in C57/BL mice. KTM2 osteosarcoma lung metastases were also reduced in Balb/C mice following CXCR4 inhibition. All three peptides significantly inhibited subcutaneous growth of SN12C-EGFP renal cancer cells. A novel class of CXCR4 inhibitory peptides was discovered. Three peptides, R, I and S inhibited lung metastases and primary tumor growth and will be evaluated as anticancer agents.

## Introduction

Chemokines are a large family of 8 to 12 kDa peptides that serve as chemoattractants for cellular activation, differentiation and trafficking. To date, about 50 chemokines have been identified in humans, and these have been grouped into four families - CXC, CC, CX3C, and XC - based on the arrangement of cysteine residues involved in the formation of disulfide bonds [1]–[3]. The biological activities of chemokines are exerted via seven transmembrane domain G-protein coupled chemokine receptors having long disordered N and C-terminal regions and three extracellular loops and three intracellular loops. The chemokine CXCL12 (stromal cell-derived factor-1α) binds to the CXCR4 and CXCR7 receptors, initiating divergent signaling pathways that result in chemotaxis, cell survival and/or proliferation, increased intracellular calcium and transcription of genes critical for cell inflammation and cancer metastases [4], [5]. CXCR4 receptor activation is mediated by coupling to an intracellular heterotrimeric G-protein associated with the inner surface of the plasma membrane [4], [5]. Although it was initially thought that CXCR4 only transduces through an intracellular heterotrimeric G-protein subunit Gαi [4], recent evidence suggests CXCR4 involves Gαq, Gαo, and Gαs and thus activates different downstream pathways. A newly discovered receptor, CXCR7, binds CXCL12 with higher affinity than CXCR4 [6], [7] and regulates CXCR4 function [8]. While CXCR4 activity is primarily G-protein mediated, the transduction pathway originating from the CXCR7 receptor seems to involve the β-arrestin pathway and is G-protein independent [9], [10].

The CXCL12/CXCR4 axis function in adults is integral to lymphocyte trafficking and to the retention and homing of hematopoietic stem cells in the bone marrow microenvironment [11], [12]. In cancer, CXCR4 expression was first correlated with the metastatic capability of breast and melanoma cancer cells ([5]); then a direct correlation between receptor upregulation and tumor progression, neovascularization, invasion and metastasis was demonstrated [13]–[20]. CXCL12 is constitutively expressed in lung, liver, skeletal muscle, brain, kidney, heart, skin and bone marrow and is induced in tissue damage such as myocardial infarction, limb ischemia, toxic liver damage, excessive bleeding, total body irradiation, and chemotherapy [17]–[20]. It has also been implicated in the recruitment of bone marrow derived cells (BMDCs) into tumors [20], [21].

As result of its pleiotropic role in tumor development, the CXCR4-CXCL12 pathway is considered an important potential cancer therapeutic target. Plerixafor (previously known as AMD3100) is a CXCR4 antagonist that has provided proof of concept for inhibition of the pathway. Mobilization with G-CSF plus Plerixafor reduces the incidence of failure to collect the minimum number of CD34 stem cells necessary for autologous stem cell transplantation. Consequently, Plerixafor in combination with G-CSF has FDA approval for hematopoietic stem cell mobilization in patients with non-Hodgkin lymphoma and multiple myeloma [22]. Plerixafor, a metal-chelating bicyclam, has been reported to cause cardiotoxicity and other adverse events, leading to the consensus opinion that it is not a suitable agent for long-term use as an anticancer agent [23]–[24].

To develop new CXCR4 antagonists suitable for anticancer therapy, a ligand-based approach was taken. Like other members of the chemokine family, CXCL12 has a short N-terminal region, an ∼10-amino acid loop that follows the CC/CXC motif (*N-loop*), a large well-folded core characterized by a three-strand antiparallel β-sheet, a C-terminal α-helix and N- and C-terminal distal regions [25], [26]. Searching for short structural motifs in the ligand receptor-binding region, a three-residue segment was identified in CXCL12 that was similar to, in reverse order, a peculiar inhibitory chemokine secreted by herpes virus 8 (HHV8) known as vMIP-II. The motif Ar1-Ar2-R, where Ar is an aromatic residue [27]–[31], constitutes the core of a cyclic peptide library that was tested in *in vitro* and *in vivo* for its ability to inhibit CXCR4 function [32].

## Materials and Methods

### Synthesis/Design Methods

The 19-membered library, consisting of different cyclic peptides, was synthesized on solid phase by using Fmoc chemistry standard protocols. All compounds were obtained in good yield and with high purity grade (>95%) after RP-HPLC purification. They were fully characterized for their identity by mass spectrometry. Starting structures were modeled from the experimental CXCL12 backbone conformation of the R-Ar2-Ar1 motif by mutating the preceding and following residues into cysteine residues, while the sequence sense was alternatively derived from vMIP-II or CXCL12. The designed peptides were energy minimized and then subjected to molecular dynamics in solution.

### Cell Culture

PES 43 human melanoma cells were grown at 37°C in 5% CO_2_ in IMDM with 10% fetal bovine serum (FBS). CCRF-CEM human T-Leukemia cells were grown in RPMI-1640 with 10% FBS and 2 mM glutamine. B16-CXCR4 murine melanoma cell line were transfected with pYF1-fusin plasmid containing CXCR4 gene (kindly provided by Dr Aloj, NCI “Pascale”, Naples, Italy) SN12C human renal cancer cells were transfected with pEGFP-1 (BD biosciences Clontech). The transfected cells were grown at 37°C in 5% CO_2_ in IMDM with 10% FBS and 2 mM glutamine 50 µg/mL penicillin, 50 µg/mL streptomycin and 100 µg/mL G418. B16 and SN12C cells were transfected with CXCR4 according to FuGEN 6 protocol (Roche Applied Science, Indianapolis, IN). All the tested cell lines were morphologically identified monthly.

### Migration Assay and Wound Closure

Migration was assayed in 24-well Transwell chambers (Corning Inc., Corning, NY) using inserts with an 8-µm pore membrane. Membranes were precoated with collagen (human collagen type I/III) and fibronectin (20 µg/mL each). PES43 cells were placed in the upper chamber (2.5×10^5^ cells/well) in IMDM containing 1% BSA (migration media) in the presence of AMD3100 or peptides; 100 ng/mL CXCL12 was added to the lower chamber. After 16 h incubation, cells on the upper surface of the filter were removed using a cotton wool swab; migration of cells in migration media alone was compared with migration in media containing CXCL12. The cells were counted in ten different fields (original magnification ×40). The migration index was defined as the ratio between migrating cells in the experimental group and migrated cells in the control group. For the wound closure assay, tested cells were allowed to reach confluence in six-well plates containing serum-depleted growth medium and scratched with pipette tips to make wounds. The wound closure was observed microscopically 6 hours post-wounding (OKO Time Lapse).

### CXCR4 Binding

CXCR4 binding was evaluated as previously described [33]. Briefly 5×10^5^ CCRF–CEM cells were pre-incubated with 10 µM peptides or AMD3100 in binding buffer (PBS 1× plus 0.2% BSA and 0.1% NaN_3_) for 30 minutes at 37°C, 5% CO2 and then labeled for 30 minutes with anti-CXCR4 PE-antibody (FAB170P, clone 12G5, R&D Systems, Minneapolis, MN, USA). The cells were analyzed by FACS Canto II cytofluorometer (Becton Dickinson Immunocytometry Systems, Mountain View, CA, USA). To evaluate the specific peptide binding to CXCR4, the experiments were also conducted in MCF-7 cell line, CXCR7 overexpressing cells using anti-CXCR7 antibody (R&D FAB4227A clone 11G8) and in COLO205, human colon cancer cells, CXCR3 overexpressing cells, using anti-CXCR3 antibody (BD Pharmingen 560831 clone 1C6/CXCR3).

### Calcium Mobilization Assay

For the calcium efflux studies a 2 mg/mL stock solution of Fluo-3 acetoxymethylester (Invitrogen, Carlsbad, CA) in anhydrous DMSO was used. A 20% w/v stock solution of the detergent pluronic acid F-127 (Invitrogen, Carlsbad, CA) was also prepared in DMSO. CCRF–CEM cells were resuspended in cell loading buffer (PBS, 1% FCS, 1 mmol/L MgCl_2_, and 1 mmol/L CaCl_2_) at 5×10^5^ cells/mL. Fluo-3AM and Pluronic acid were added to each sample to increase Fluo-3AM solubility and improve dye loading into the cells. Baseline calcium efflux was established, then chemokines were added as indicated and chemokine-induced calcium efflux was measured.

### Immunoblotting

Cells were homogenized in lysis buffer (40 mM Hepes pH 7,5, 120 mM NaCl, 5 mM MgCl_2_, 1 mM EGTA, 0,5 mM EDTA, 1% Triton X-100) containing protease (Complete Tablets- EDTA-free, Roche) and phosphatase inhibitors (20 mM α-glycerol-3-phosphate, 2,5 mM Na-pyrophosphate). The following primary antibodies were used: anti-p-ERK (sc7383, Santa Cruz Biotechnology, Inc., Santa Cruz, CA, USA) and anti-ERK2 (sc 154G, Santa Cruz Biotechnology CA, USA). P-Erk induction was plotted as ratio P-Erk in the presence of CXCL12 (100 ng/ml)/P-Erk in serum free.

### *In Vivo* Assays

All mice were obtained from Harlan (Bar Harbor, ME, USA) and their care was in accord with institutional guidelines. Twenty-five 6-8-week-old female C57BL/6 mice were inoculated into the tail vein with 5×10^5^ B16–CXCR4 cells pre-treated for 30 minutes with peptide S (10 µM), peptide R (10 µM), peptide I (10 µM), or AMD3100 (10 µM). Six hours later intraperitoneal (IP) treatment started with AMD3100 (1.25 mg/kg) or each peptide (2 mg/kg) in sterile PBS once a day for 10 days. A week later the mice were sacrificed. The experiment was repeated three times. The lung metastases assay was also conducted with K7M2 murine osteosarcoma cells. Twenty-five 6-8-week-old female Balb/c mice were injected via tail vein with 2.5×10^5^ K7M2 cells pre-treated for 30 minutes with AMD3100 (10 µM), peptide S (10 µM), peptide R (10 µM), peptide I (10 µM) and animals were treated IP daily for 15 days with 2.5 mg/kg for AMD3100 group and 10 mg/kg for each peptide groups. A week later the mice were sacrificed. Xenograft tumor growth was evaluated using pEGFP-SN12C cells in exponential growth phase subcutaneously injected into the right flank of twenty-five 6-8-week-old female CD1 nude mice. 2×10^6^ SN12C-pEGFP were injected in the right flank of each mouse and treated with AMD3100 (1.25 mg/kg) and peptides (2 mg/kg) for 10 days ip. A week later mice were euthanized and tumor volume evaluated through Leica MacroFluo fluorescence stereomicroscope (Leica microsystem, Houston, TX). The experiment was repeated three times. The Istituto Nazionale per lo Studio e la Cura dei Tumori, Fondazione Giovanni Pascale Independent Etical Comettee approved the study (CEI/600/12).

## Results

### Rational Design of a Novel Class of Cyclic Peptides that Inhibit CXCR4

To develop new CXCR4 antagonists suitable for anticancer therapy, a ligand-based approach was employed. Previous NMR studies on the vMIP-II N-terminal tail showed that a structured motif encompassing tryptophan 5 to proline 8 (W5-P8) preserved its conformation in the intact protein, making this motif a promising candidate scaffold to design short CXCR4-ligand peptides. A three-residue motif, Trp-His-Arg (Ar1-Ar2-R), was identified in the vMIP-II N-terminus [25]–[31] and after comparison with the CXCL12 structure, a similar motif was identified in CXCL12, but in the N-loop and with the sequence reversed, Arg-Phe-Phe (R-Ar1-Ar2) [30]. Consequently, the two motifs were used as templates to design cyclic peptides with the structure C-Ar1-Ar2-R-C and C-R-Ar1-Ar2-C, with the cysteines at each end in a disulfide-bridge to stabilize the structure and provide protection from proteases. In addition, peptides were either elongated at their C-termini or, after sequence-reversal, at their N-termini, so as to mimic another possibly conserved basic residue motif. The peptide series shown in Table 1 was synthesized to undergo biochemical and biological characterization (Italian Patent n° MI2010A000093; International Patent n° WO2011/092575 A1) [32]. Peptides were CXCL12-mimetic (R, S and T, shown in bold) and v-MIP-II mimetic (I and all others). Representative structures for Peptides R, I, O and D are showed in **Figure S1**.

**Table 1:** Peptide sequences, one-letter code names and shortened sequence notations.

Sequence*	Code	Shortened sequence**
**Arg-Ala-[Cys-Arg-Phe-Phe-Cys]**	**R**	**RACRFFC**
[Cys-Phe-Phe-Arg-Cys]	G	CFFRC
[Cys-Phe-Phe-Arg-Cys]-Ala-Arg	O	CFFRCAR
Acetyl-[Cys-Phe-Phe-Arg-Cys]	A	Ac-CFFRC
[Cys-Phe-Phe-Arg-Cys]-Amide	H	CFFRC-Nam
Acetyl-[Cys-Phe-Phe-Arg-Cys]-Amide	B	Ac-CFFRC-Nam
**Arg-Ala-[Cys-Arg-His-Trp-Cys]**	**S**	**RACRHWC**
**[Cys-Trp-His-Arg-Cys]**	**I**	**CWHRC**
[Cys-Trp-His-Arg-Cys]-Ala-Arg	P	CWHRCAR
Acetyl-[Cys-Trp-His-Arg-Cys]	C	Ac-CWHRC
[Cys-Trp-His-Arg-Cys]-Amide	L	CWHRC-Nam
Acetyl-[Cys-Trp-His-Arg-Cys]-Amide	D	Ac-CWHRC-Nam
**Arg-Ala-[Cys-Arg-Tyr-Trp-Cys]**	**T**	**RACRYWC**
[Cys-Trp-Tyr-Arg-Cys]	M	CWYRC
[Cys-Trp-Tyr-Arg-Cys]-Ala-Arg	Q	CWYRCAR
Acetyl-[Cys-Trp-Tyr-Arg-Cys]	E	Ac-CWYRC
[Cys-Trp-Tyr-Arg-Cys]-Amide	N	CWYRC-Nam
Acetyl-[Cys-Trp-Tyr-Arg-Cys]-Amide	F	Ac-CWYRC-Nam
[Cys-Trp-Trp-Arg-Cys]	V	CWWRC

* Square brackets indicate cyclization via disulfide-bridge.

** Ac = Acetyl,Nam = amide NH_2_.

### Peptides R, I, S and T Impair the Binding of 12G5 to CXCR4

**Figure 1:**
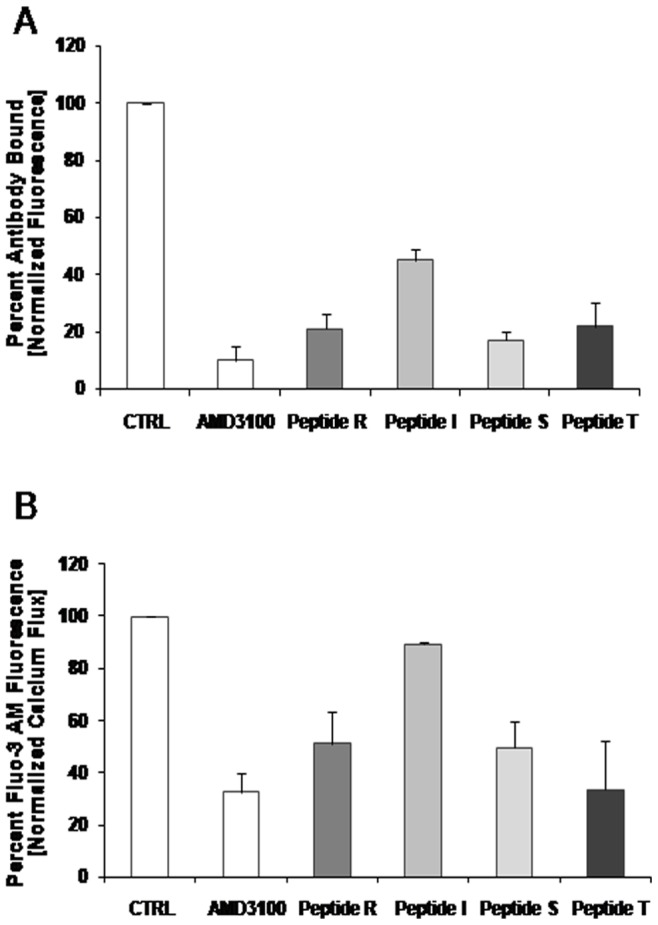
Peptides R, I, S, T impair 12G5-CXCR4 binding and CXCL12-induced Ca^2+^ influx in CCRF–CEM cells. (A) The amount of peptides or AMD3100 bound was assessed indirectly by flow cytometry using the PE-labelled anti-CXCR4 antibody (clone 12G5). Results are expressed as the percent antibody bound. (B) The Ca^2+^ influx assay was performed using CCRF-CEM cells and Fluo-3 AM calcium indicator. CCRF-CEM cells were incubated 30 minutes at 37°C with Fluo-3 AM and 15 minutes with AMD3100 (10 µM)/peptides (10 µM) and then treated with CXCL12. Results are expressed as the percent of Fluo-3 AM fluorescence in presence of CXCL12 alone. Each peptide was tested in at least three different experiments. Double tailed T-Test was used for statistical analyses. Differences were considered significant at *P*<0.05 compared to control. Control (CTRL).

An initial biological characterization regarded possible peptide toxicity. Peptides R, I and S were evaluated for cytotoxicity on several human cancer cell lines and showed no toxicity (**Figure S2**). The anti-CXCR4 cyclic peptides were first evaluated for their ability to inhibit the association of the 12G5 anti-CXCR4 antibody with the CXCR4 receptor [22]. CCRF-CEM cells were incubated with the anti-CXCR4 cyclic peptides (10 µM) or with the CXCR4 inhibitor, AMD3100 (10 µM) for 30 minutes. **Figure 1A** shows the effect of the peptides R, I, S, and T (10 µM) on the binding of 12G5 to CXCR4. Peptides R, S, and T, seven-residue CXCL12-mimetics with elongation at the N-terminus, reduced the association of the 12G5-CXCR4 antibody to the receptor to 21, 17 and 22% of control, respectively, a level of inhibition comparable to that obtained with AMD3100; the five-residue peptide I, a v-MIPII-mimetic, inhibited the association of the 12G5-anti-CXCR4 antibody to 45% of control. Since CXCL12 also binds CXCR7, its association with the CXCR7 receptor in the CXCR7 expressing cell line, MCF7, was evaluated in the presence of peptides R, I, S and T. The unrelated CXCR3-expressing cell line, COLO205, was also evaluated. **Figures S3A-C** show that peptide S inhibited 12G5-CXCR4 binding to CXCR4 comparably to AMD3100 while the binding to CXCR3 or CXCR7, which was inhibited by the specific ligand, CXCL11, was unaffected by peptides R, I and S.

### Peptides R, I, S and T Inhibit Calcium Release

As seen with other G-protein coupled receptors (GPCR), CXCR4 activation results in recruitment of Gq protein that in turn leads to PLCγ activation and Ca^2+^ influx. [4]. The effect of peptides R, I, S, and T (10 µM) on CXCL12-induced calcium release was evaluated using CCRF-CEM cells. The cells were incubated in the presence of peptide and then loaded with Fluo-3AM in the presence of F-127 Pluronic Acid. As shown in **Figure 1B** **,** the peptides clearly inhibited CXCL12 induced calcium efflux: R (51.25% of control), S (49.75%), and T (33.65%). Minimal effect on calcium efflux was observed with Peptide I. A representative experiment with peptide T is shown in **Figure S3D**.

### Peptides R, I, S, and T Inhibit CXCL12-dependent Cell Migration, Wound Healing and P-ERK Induction

**Figure 2:**
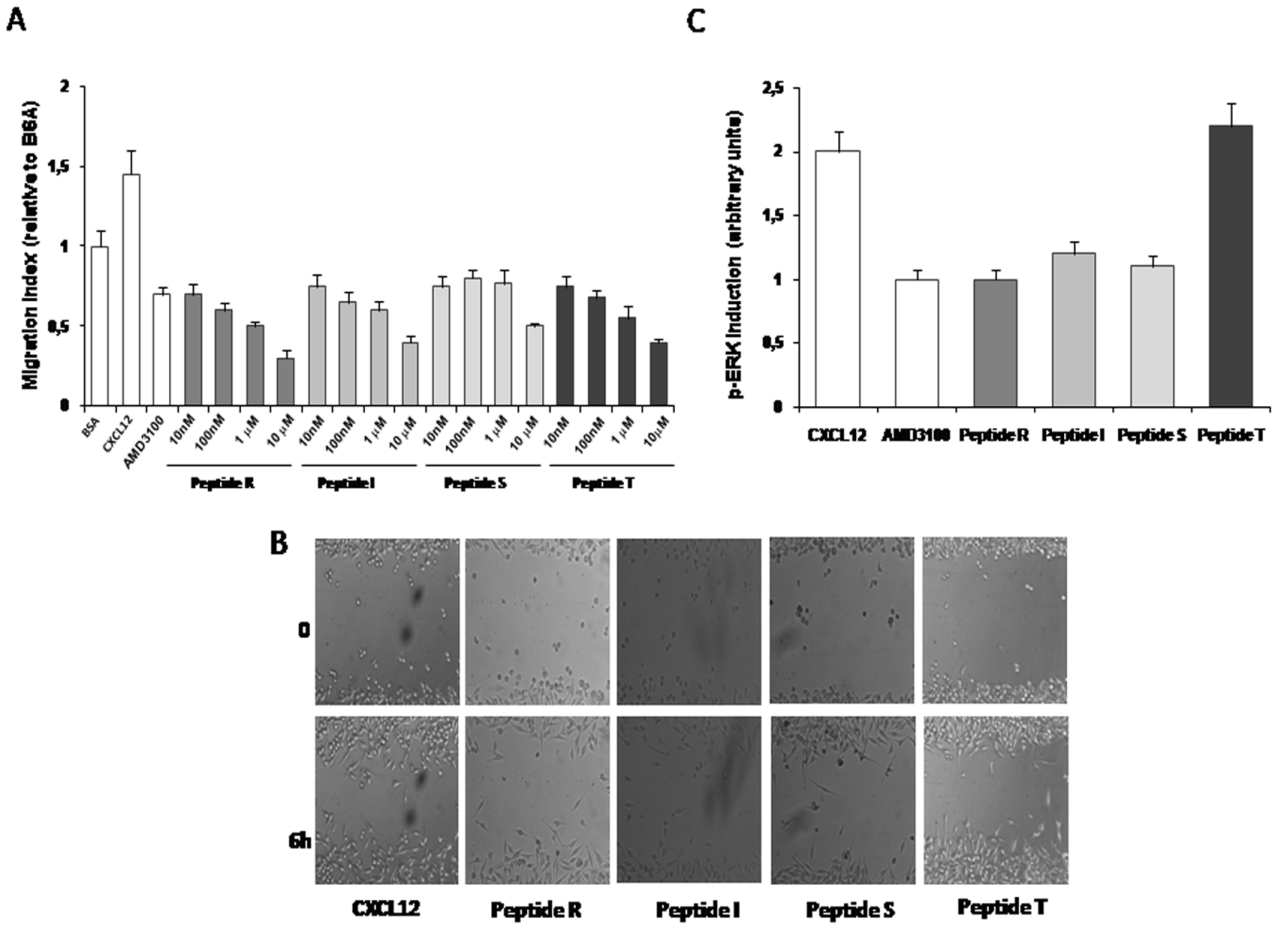
Peptides R, I, S, T inhibit CXCL12 dependent cell migration, wound healing and p-ERK induction. Experiments were conducted using PES43 cells. (A) Migration was assayed in 24-well Transwell chambers using inserts with 8-µm pore membrane. PES43 cells were placed in the upper chamber (2.5×10^5^ cells/well) in IMDM containing 1% BSA (migration media) in the presence of AMD3100 (10 M) or peptides at several concentrations (10 nM, 100 nM, 1 µM, 10 µM); 100 ng/mL CXCL12 was added to the lower chamber. Migrated cells on the lower surface were fixed, stained with H&E and counted microscopically. The results are expressed as the migration index relative to migration in presence of BSA alone. (B) Delay in wound healing after 6 hours in presence of peptides R, I, S ant T compared with CXCL12. Images were acquired with OKO Time Lapse. (C) Effect of peptides R, I, S, and T on CXCL12 p-ERK induction at 5 minutes. PES43 cells were serum-starved and incubated with CXCL12 (100 ng/ml) alone or in presence of AMD3100/peptides. Each peptide was tested in at least three different experiments. Double tailed T-Test was used for statistical analyses. Differences were considered significant at *P*<0.05 compared to control.

Functional CXCR4 transduces signals that activate chemotaxis and phosphorylation of ERK through multiple and complex pathways, such as Gαi-AC, β-arrestin-RAF and Gαq-PI3K [4]. To further characterize the effect of peptides R, I, S, and T on CXCR4 function, CXCL12-dependent p-Erk induction and migration were analyzed in PES43, a human melanoma cell line previously demonstrated to exhibit high levels of a functional CXCR4 [34]. **Figure 2A** shows that peptides R, I, S, T inhibited migration at concentrations as low as 10 nM. Also, given that CXCL12 primarily is a lymphocyte chemoattractant, effects of the peptides R, S and I were evaluated on CCRF-CEM human T-Leukemia cells migration. As shown in **Figure S4** Peptide R inhibited CCRF-CEM human T-Leukemia cells migration in a dose-dependent manner. **Figure 2B** demonstrates that the peptides R, I, S and T delayed CXCL12-mediated wound healing in PES43 human melanoma cells. **Figure 2C** depicts the inhibition of CXCL12 induced p-ERK by peptides R, I, S, T. Like AMD3100, peptides R, I, and S (but not peptide T), reduced CXCL12-mediated p-ERK induction after 5 minutes. A representative experiment with peptide R is shown in **Figure S5**. In the absence of CXCL12, the peptides had a negligible effect on P-ERK induction. The in vitro evaluation of the CXCR4 inhibitory efficacy resulted in the selection of four peptides R, S, T and I that were consistently impairing CXCR4 function although performing differently in the tested assays (Peptide R revealed the best efficacy in inhibiting CXCL12 dependent migration, P-Erk induction and wound healing; Peptide S efficiently competed with the anti CXCR4 antibody binding and Peptide I showed the best efficacy in inhibiting Calcium efflux ). This is not surprising since the 12G5-anti CXCR4 antibody binding versus inhibition in migration, calcium efflux or P-Erk induction- CXCL12 measured really different properties that involves different sites of interaction.

### Peptides R, I and S Inhibited Murine Lung Metastases and Human Primary Tumor Growth

**Figure 3:**
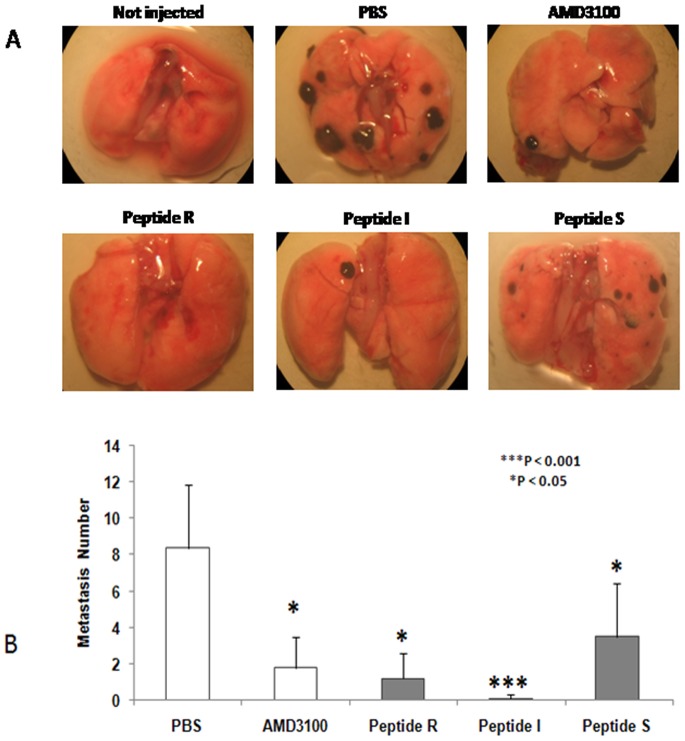
Peptides R, I and S inhibit murine melanoma lung metastases. (A) B16-CXCR4 tumor cells pre-treated for 30 minutes with AMD3100 (10 µM), peptide R (10 µM), peptide I (10 µM), or peptide S (10 µM) were inoculated into the tail vein of C57/B female mice and the mice were further treated intraperitoneally for 10 days with 1.25 mg/kg AMD3100, or 2 mg/kg peptide R, peptide I or peptide S. Gross examination of representative lungs; (B) Graphical representation of the number of lung metastases in treated mice. Double tailed T-Test was used for statistical analyses. The experiments were repeated three times.

To evaluate the potential of the peptides for clinical development, the efficacy of peptides R, I and S was evaluated in *in vivo* lung metastasis models [35], [36]. While these models are often used to evaluate inhibition of metastases, we used the assays to detect the ability of the peptides to inhibit lung colonization after the injection of tumor cells. Peptide T could not be evaluated *in vivo* because of poor solubility. 5×10^5^ B16 melanoma cells transduced with CXCR4 were pre-treated with peptides R, I or S (10 µM) or AMD3100 (10 µM) and inoculated into the tail vein of C57/BL mice. Mice were then further treated once a day for 10 days with 2 mg/kg peptide R, I, or S or with 1.25 mg/kg AMD3100 intraperitoneally (i.p.). As shown in **Figure 3** **,** gross inspection of the lungs of treated mice showed a marked decrease in secondary lesions. Compared to an average of 8.4±3.49 metastases in the control, peptides R, I and S reduced the number of metastases 7-, 41.5-and 2,8 fold to 1.2±1.4, 0.2±0.44 and 2.96±2.39, respectively, while AMD3100 reduced metastases 5.6 fold to 1.5±2.9.

**Figure 4:**
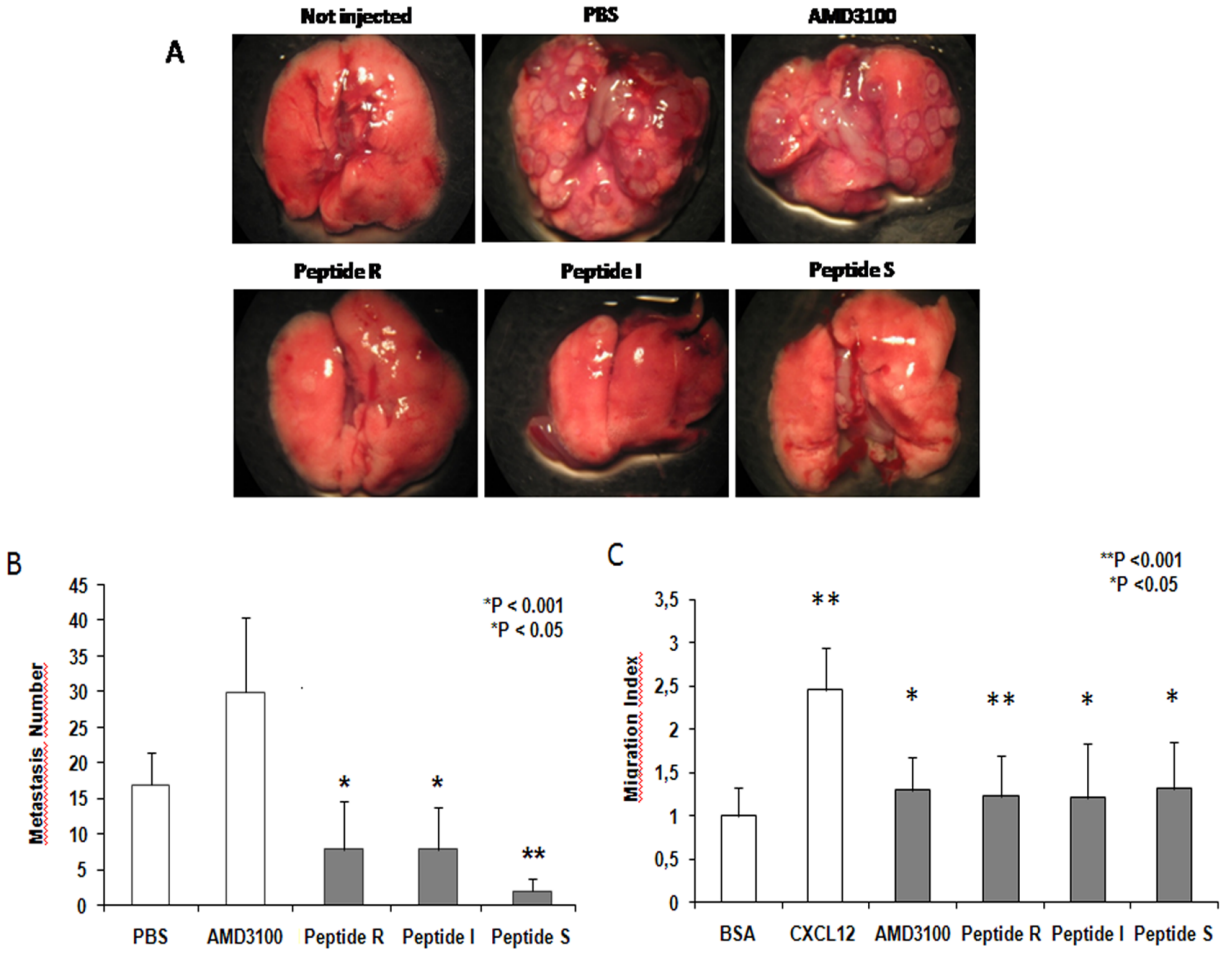
Peptides R, I and S inhibit murine osteosarcoma lung metastases. (A) Twenty-five 6-8-week-old female Balb/c mice were injected via tail vein with 2.5×10^5^ K7M2 cells pre-treated for 30 minutes with AMD3100 (10 µM), peptide R (10 µM), or peptide I (10 µM) or peptide S (10 µM). The animals were then further treated intraperitoneally for 15 days with 2.5 mg/kg AMD3100 or 10 mg/kg peptide R, peptide I or peptide S. (B) Graphical representation of the number of lung metastases in treated mice. Double tailed T-Test was used for statistical analyses. The experiments were repeated three times.

To further evaluate the extent of peptide efficacy, a similar *in vivo* experiment was performed in a syngenic K7M2 osteosarcoma model in Balb/C mice (36]) (**Figure 4**). Again, marked reduction in lung metastases formation was observed when cells were pretreated with the peptides and then animals injected daily. As shown in the lower right panel of **Figure 4** **,** peptides R, I and S also reduced K7M2 *in vitro* migration.

**Figure 5:**
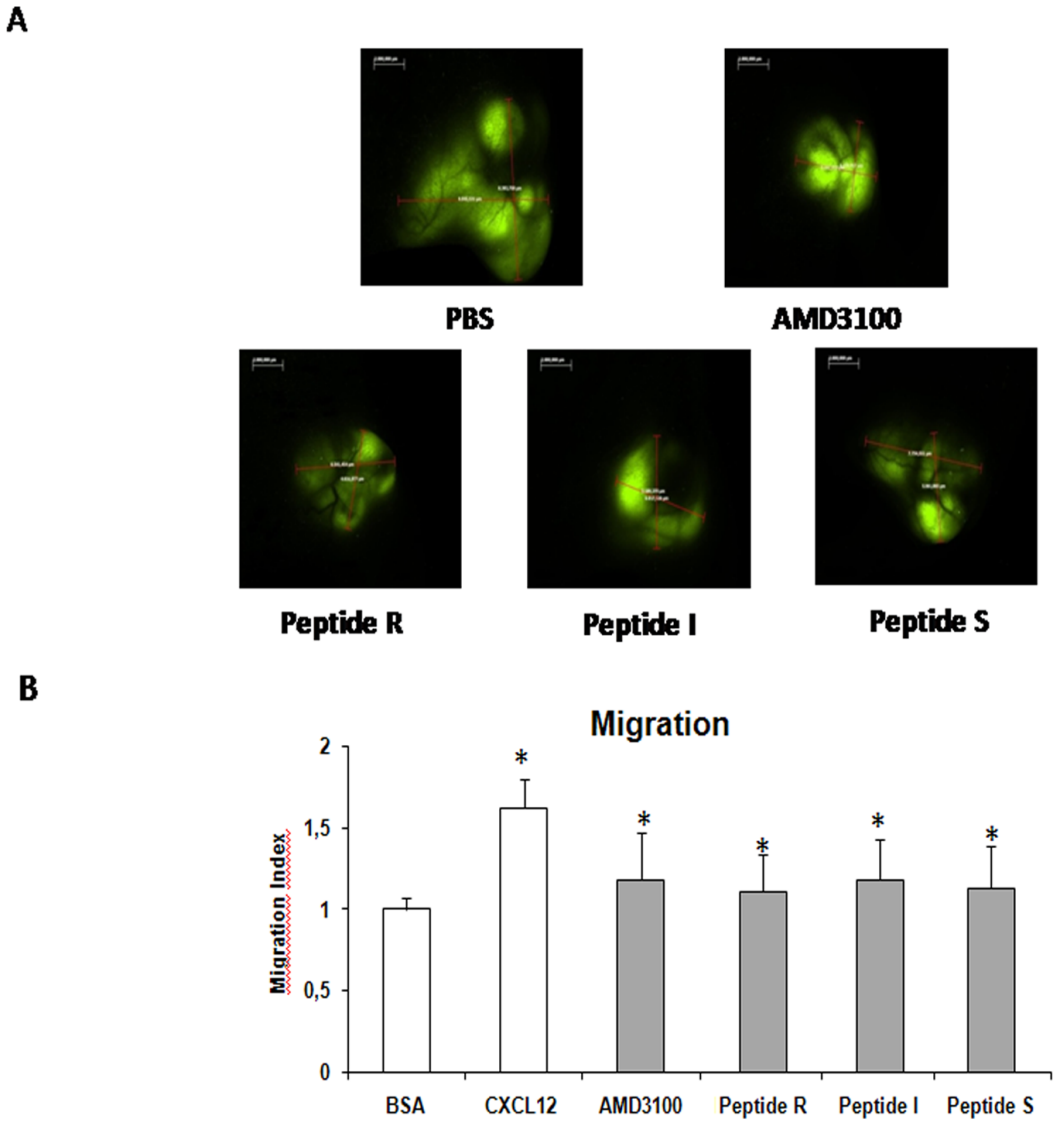
Peptides R, I, and S inhibit the growth of primary human SN12C-pEGFP tumors. 6 to 8-week-old female CD1 nude mice were injected subcutaneously with 2×10^6^ pEGFP-SN12C cells pre-treated with 10 µM AMD3100 or 10 µM of each peptide. Beginning the following day mice were further treated intraperitoneally for 10 days with 1.25 mg/kg AMD3100 or 2 mg/kg of peptides R, I or S. A week later mice were euthanized and tumor volume evaluated directly from the flank of mice using a Leica MacroFluo fluorescence stereomicroscope (Leica microsystem, Houston, TX) (A). The experiments were repeated three times. B. SN12C-PEGFP cells migration toward CXCL12 in the presence of AMD3100 or Peptide R, S and I.

Since CXCR4 inhibition was reported to interfere with tumor cell growth [37], we evaluated the effects of peptides R, I, and S on the growth of subcutaneous human renal SN12C cells engineered to express green fluorescence protein (GFP). As shown in **Figure 5A** treatment with peptides R, I, or S or with AMD3100 reduced the growth of SN12C-GFP cells: R (average 47.00±16.88 mm^3^), I (average 39.19±25.8 mm^3^), S (average 44.51±26.4 mm^3^), or AMD3100 (average 35.53±10.42 mm^3^), *versus* control (average 65.97±35.1 mm^3^). **Figure 5B** shows the corresponding H&E stain. In addition, as shown in the lower panel of **Figure 5C** peptides R, I and S inhibited CXCL12-induced SN12C-GFP migration.

## Discussion

In this manuscript we describe a new class of ligand based, cyclic peptide inhibitors of CXCR4. Three novel cyclic peptides are shown to impair CXCR4 function *in vitro* and *in vivo*. Nineteen peptides based on an Ar1-Ar2-R motif, identified in vMIP-II and found to be present in the opposite orientation as R-Ar1-Ar2 in the N-loop of CXCL12, were initially assessed. The nineteen peptides were based on the identified motif, in both sequence sense, plus elongation at their C- or - N-termini or modification of aromatic residues. The entire peptide library was then screened in vitro for CXCR4 interfering activity. Based on the rational design, while seeking for CXCR4 antagonistic peptides, we were not expecting all of them to work. Thus peptides showing concomitant antagonistic activity in the four in vitro assays (competition with anti CXCR4 antibody binding, ligand dependent migration, calcium efflux and P-Erk induction) were further pursued.

To identify the best CXCR4 inhibitors suitable for anticancer therapy peptides R, I, S and T were selected for in vivo evaluation. Solubility limitations prevented the evaluation of peptide T *in vivo*, but peptides, R, I and S, reduced lung metastases in mice injected with B16-CXCR4 mouse melanoma cells and K7M2 mouse osteosarcoma cells. In addition, peptides R, I and S inhibited primary tumor growth in a xenograft model of human renal cancer cells, SN12C.

CXCR4 antagonism was defined on the basis of competition with anti-12G5-CXCR4 antibody, as well as inhibition of migration, calcium flux and P-Erk induction. While the peptides were designed to block CXCR4 and they clearly inhibit its function, we recognize that the competition assays we used do not directly measure peptide binding to CXCR4.

According to the initial design, three of these peptides are CXCL12-mimetic (R, S and T) and one is vMIP-II mimetic (I); interestingly three of the four contained the RA-NH2 extension deemed crucial for binding. Theoretical complexes obtained for peptides R, I and S showed their binding sites lay entirely in the intrahelical site, similar to AMD3100 and CVX15, a cyclic peptide inhibitor of CXCR4. Although AMD3100 and peptides R, I and S share a common core of ligand-receptor interactions, they span different subsites possibly explaining differences in activities.

The CXCR4-CXCL12 axis has become more complex since the discovery of CXCR7, a deorphanized receptor that binds CXCL12. CXCL12 binds CXCR7 with an affinity even higher than CXCR4. Recent evidence shows that TC14012, a peptidomimetic inverse agonist of CXCR4, is also a CXCR7 agonist and that two structurally unrelated CXCR4 antagonists, TC14012 and AMD3100 also perform as agonists on CXCR7 [38]. Thus, CXCR4 and CXCR7 while transducing on the beta-arrestin pathway differently share similarities in their binding sites for synthetic ligands, suggesting CXCR4 inhibitors may also be active on CXCR7. Preliminary results demonstrate that peptides R, I and S do not affect CXCR7 and CXCR3 binding (See Figure S2), and none of our experiments suggest agonistic effects.

The development of CXCR4 inhibitors is of increasing interest with AMD3100 (Plerixafor) having been approved by the FDA for stem cell mobilization [21], [39]. We evaluated peptides R, I and S for their ability to mobilize hematopoietic precursors in a DBA/2 mouse model in a CXCR4 dependent manner. Preliminary evidence shows that peptides R, I, and S induce as potent stem cell and neutrophil mobilization as does AMD3100. A single treatment resulted in a rapid and dose-related 2 to 5-fold increase in neutrophils and hematopoietic stem and progenitor cells in peripheral blood. Interestingly the mobilization effect was more durable in mice treated with peptides R, I, and S compared to AMD3100 (Portella L, Proceedings AACR 2011 n. 394, manuscript in preparation).

Plerixafor, a metal chelating bicyclam, has been shown to block calcium flux inducing cardiotoxicity [22], [23], and thus is not ideal for long-term clinical use [23], [39]. Consequently, multiple CXCR4 inhibitors including peptidic molecules, other small molecules, antibodies, CXCL12-mimetics, “spiegelmers”, beta-hairpin mimetics, L-enantiomeric RNA oligonucleotides, and lipopeptid pharmacophores have been conceived for clinical development [39]. Among peptidic inhibitors, T140 derivatives from a naturally occurring horseshoe crab protein [40] and 4F-benzoyl-TN14003 (BKT-140), have demonstrated anticancer activity [41]-[43] as well as mobilizing effects [44]. A phase I/II study with BKT-140 in multiple myeloma was recently completed but results are not yet available (ClinicalTrial.gov identifier: NCT01010880, consulted 09/016/2012). Additionally, a Phase I-II clinical trial was reported for the CXCR4 inhibitor, CTCE-9908, a CXCL12 N-terminus derived peptide representing a dimerized sequence of CXCL12 amino acids 1-8 [45]–[47]. Two Phase II studies, one in renal cell carcinoma [NCT01391130] and one in small cell lung cancer [NCT 01439568] have evaluated the efficacy of the CXCR4 peptidic inhibitor LY2410924® (Eli Lilly and Company) in combination with sunitinib and carboplatin/etoposide respectively. While these clinical trials are evaluating these agents in patients with advanced cancer, the ideal setting to demonstrate the efficacy of CXCR4 inhibitors will be as metastasis-preventing agents in the adjuvant and neoadjuvant setting with tumors such as colorectal cancer [47] and in the prevention of first recurrence after radiotherapy in glioblastoma [8], [18], [48].

In summary we describe a new family of peptides that were rationally designed and not derived from the naturally occurring CXCR4 inhibitor polyphemusin-II, used as a template to design several classes of CXCR4 inhibitors. Comparative studies between the new family of peptides and Plerixafor suggest a common binding site. Peptide CXCR4 antagonist activity has been shown in the studies presented here both *in vitro* and *in vivo* and a first in human, Phase I trial is planned with peptide R in patients with advanced tumors.

## Supporting Information

Figure S1 **Representative structures for Peptides R, I, O and D are showed.**
(TIF)

Figure S2 **Peptides R, I and S were not toxic on human cancer cell lines (SN12C, RXF393, A498 and PES43).**
(TIF)

Figure S3 **Peptide S specifically inhibits 12G5-CXCR4 binding to CXCR4.** **A**. CCRF**-**CEM cells were preincubated for 30 minutes with Peptide S (10 µM) or AMD3100 (10 µM) and then incubated with 12G5 anti CXCR4 antibody. **B.** CEM cells were preincubated for 30 minutes with CXCL11 (100 nM), Peptide S (10 µM) or AMD3100(10 µM) and then incubated with anti CXCR7 antibody; C. CCRF-CEM cells were preincubated for 30 minutes with CXCL11 (100 nM), Peptide S (10 µM) or AMD3100(10 µM) and then incubated with anti CXCR3 antibody; D. Baseline calcium efflux was established, then chemokines were added as indicated and chemokine induced calcium efflux was measured. CCRF-CEM cells were preincubated for 30 minutes with CXCL12 (100 nM), Peptide T (10 µM) or AMD3100 (10 µM) or iomycin as positive control. Fluo-3AM and Pluronic acid were added to each sample to increase Fluo-3AM solubility and improve dye loading into the cells.
(TIF)

Figure S4 **Peptide R inhibited CCRF-CEM human T-Leukemia cells migration in a dose-dependent manner.**
(TIF)

Figure S5 **Peptide R inhibits the CXCL12 induced p-ERK. PES43 cell lines were serum starved for 16 hours.** Then the cells were preincubated for 30 minutes with Peptide R (10 µM) or AMD3100 (10 µM) and then treated with CXCL12 (100 nM).
(TIF)
