# Supplementary figures and images for: Preclinical Development of a Novel Class of CXCR4 Antagonist Impairing Solid Tumors Growth and Metastases
Source: PLoS One. 2013 Sep 13;8(9):e74548. doi: 10.1371/journal.pone.0074548 (PMC3772838; doi:10.1371/journal.pone.0074548)

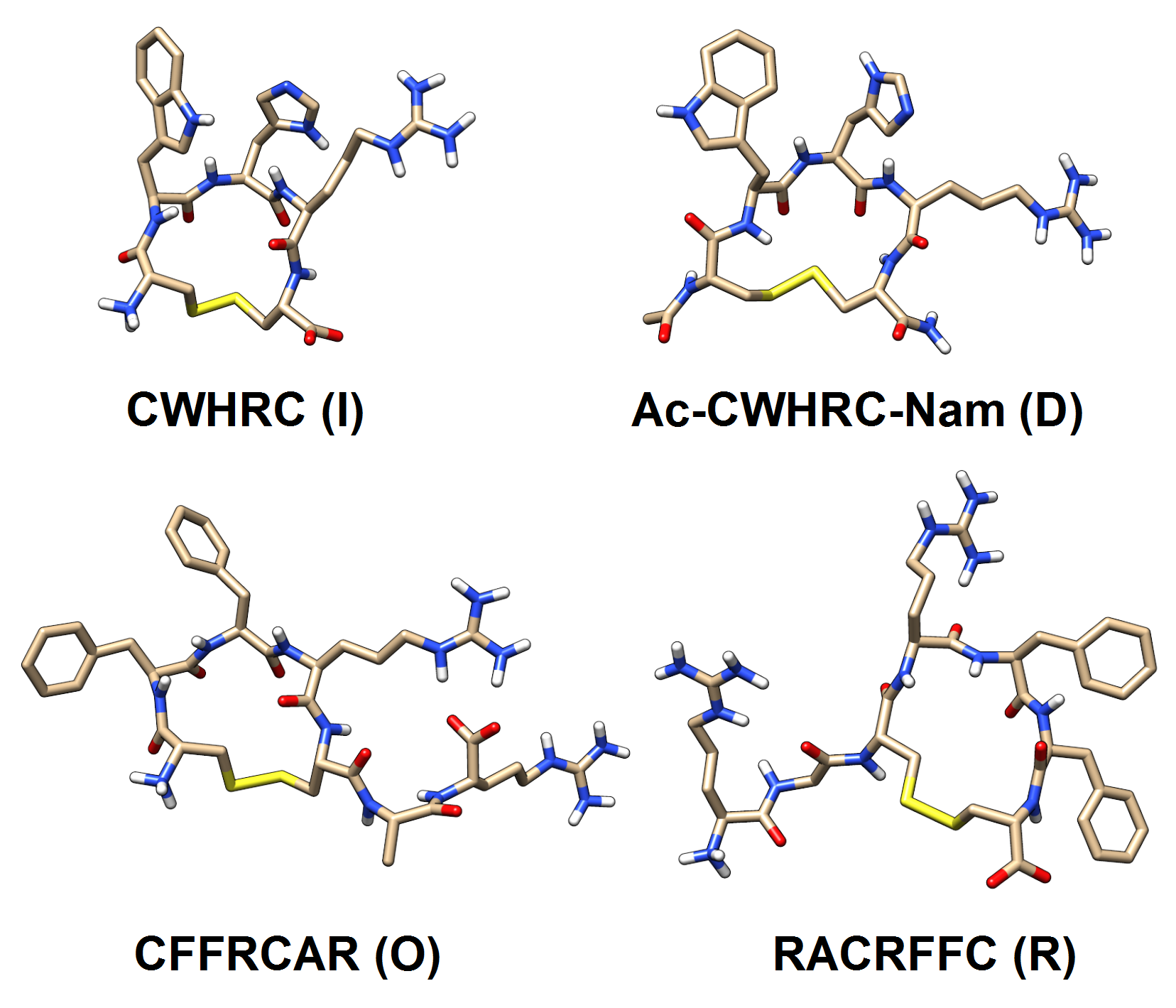

Supplement: Figure S1 — Representative structures for Peptides R, I, O and D are showed. (TIF) [file pone.0074548.s001.tif]

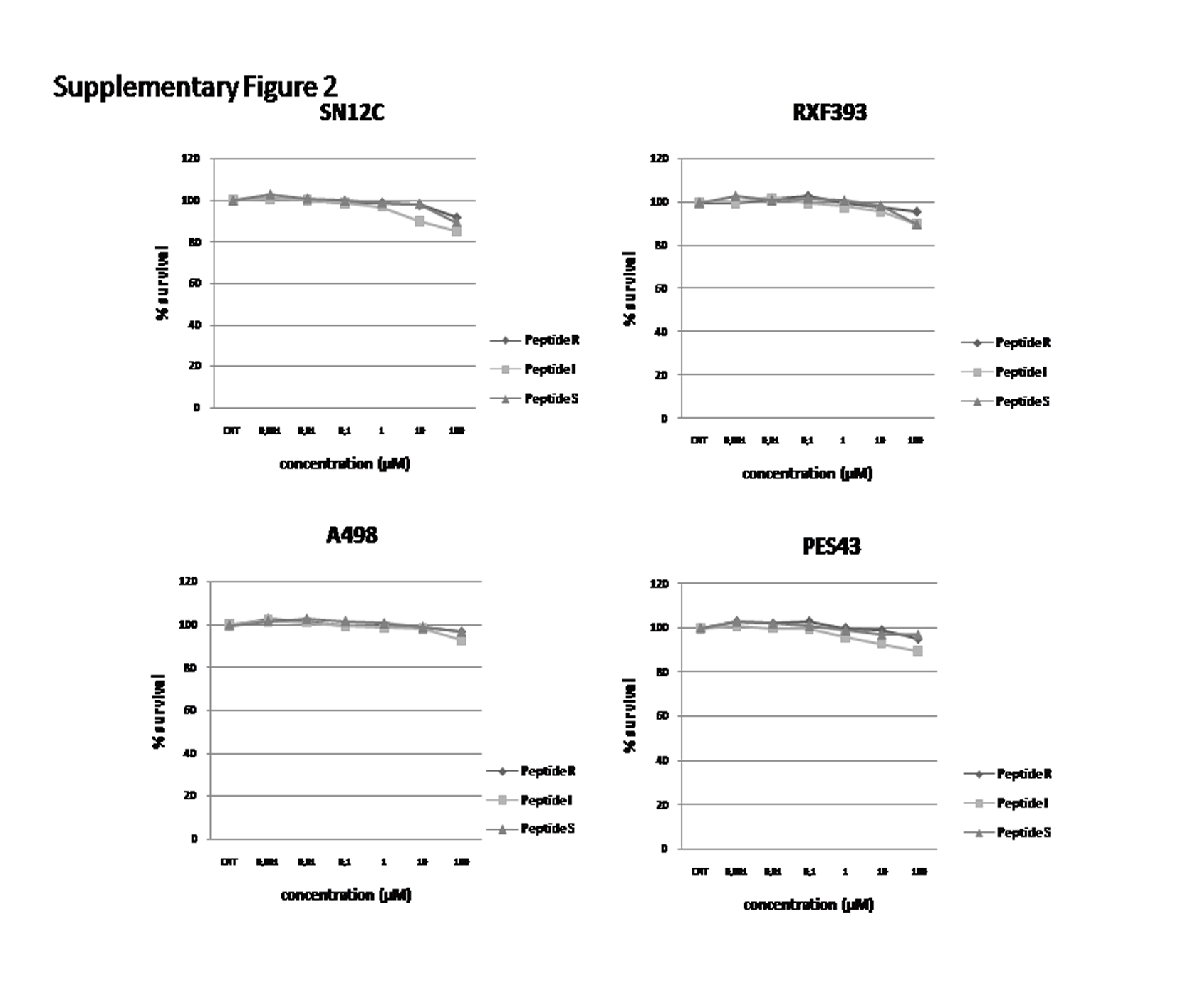

Supplement: Figure S2 — Peptides R, I and S were not toxic on human cancer cell lines (SN12C, RXF393, A498 and PES43). (TIF) [file pone.0074548.s002.tif]

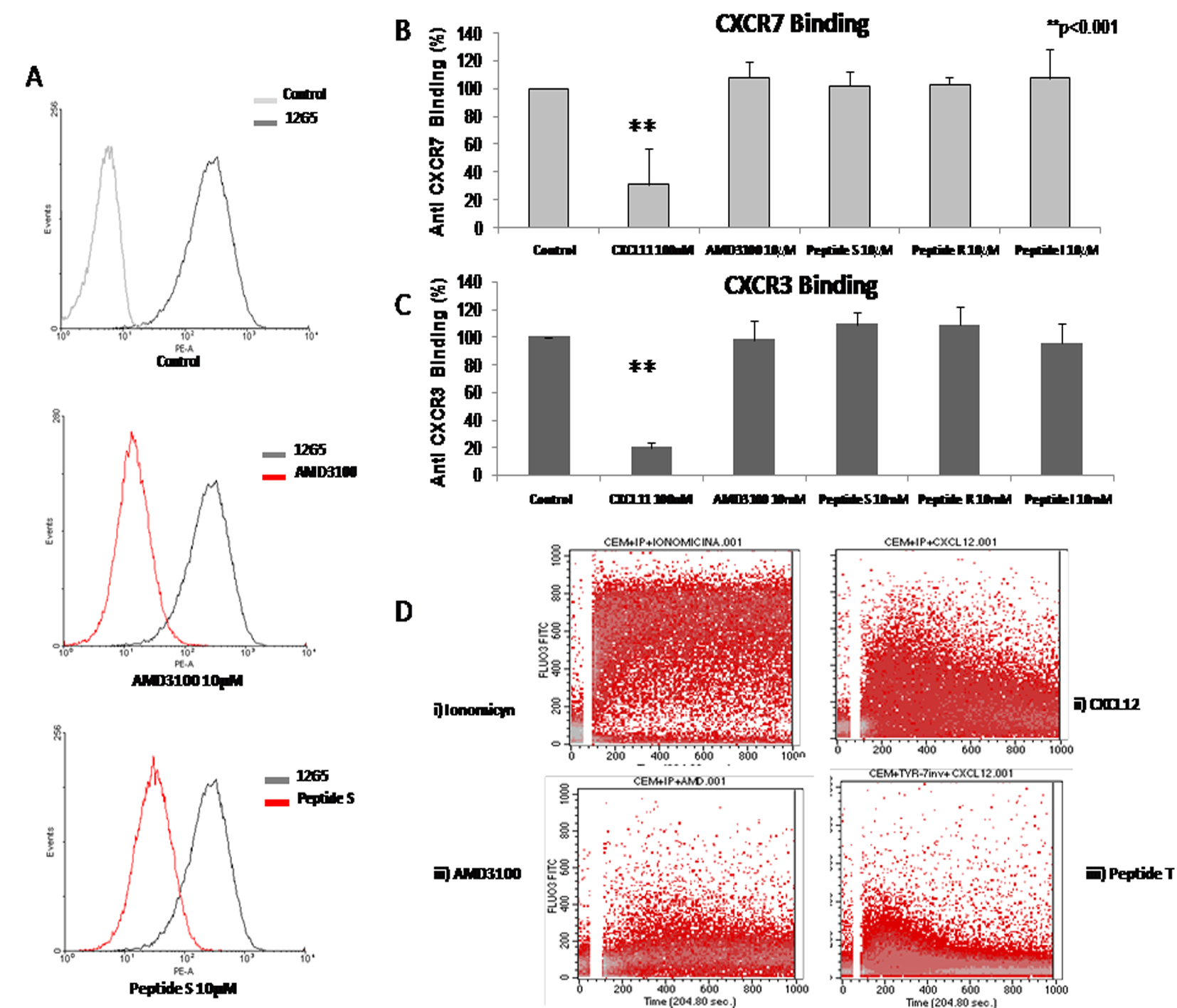

Supplement: Figure S3 — Peptide S specifically inhibits 12G5-CXCR4 binding to CXCR4. A. CCRF-CEM cells were preincubated for 30 minutes with Peptide S (10 µM) or AMD3100 (10 µM) and then incubated with 12G5 anti CXCR4 antibody. B. CEM cells were preincubated for 30 minutes with CXCL11 (100 nM), Peptide S (10 µM) or AMD3100(10 µM) and then incubated with anti CXCR7 antibody; C. CCRF-CEM cells were preincubated for 30 minutes with CXCL11 (100 nM), Peptide S (10 µM) or AMD3100(10 µM) and then incubated with anti CXCR3 antibody; D. Baseline calcium efflux was established, then chemokines were added as indicated and chemokine induced calcium efflux was measured. CCRF-CEM cells were preincubated for 30 minutes with CXCL12 (100 nM), Peptide T (10 µM) or AMD3100 (10 µM) or iomycin as positive control. Fluo-3AM and Pluronic acid were added to each sample to increase Fluo-3AM solubility and improve dye loading into the cells. (TIF) [file pone.0074548.s003.tif]

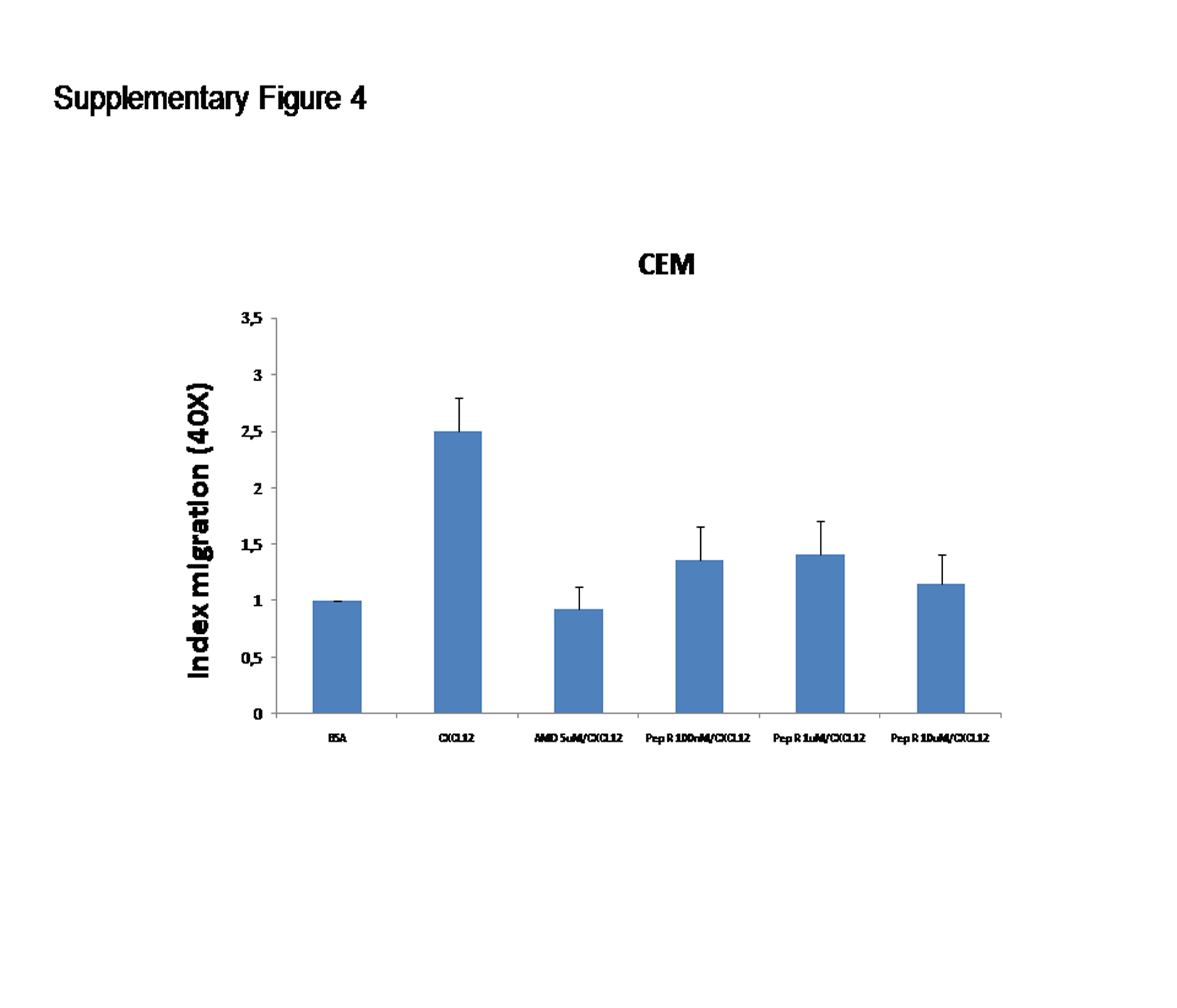

Supplement: Figure S4 — Peptide R inhibited CCRF-CEM human T-Leukemia cells migration in a dose-dependent manner. (TIF) [file pone.0074548.s004.tif]

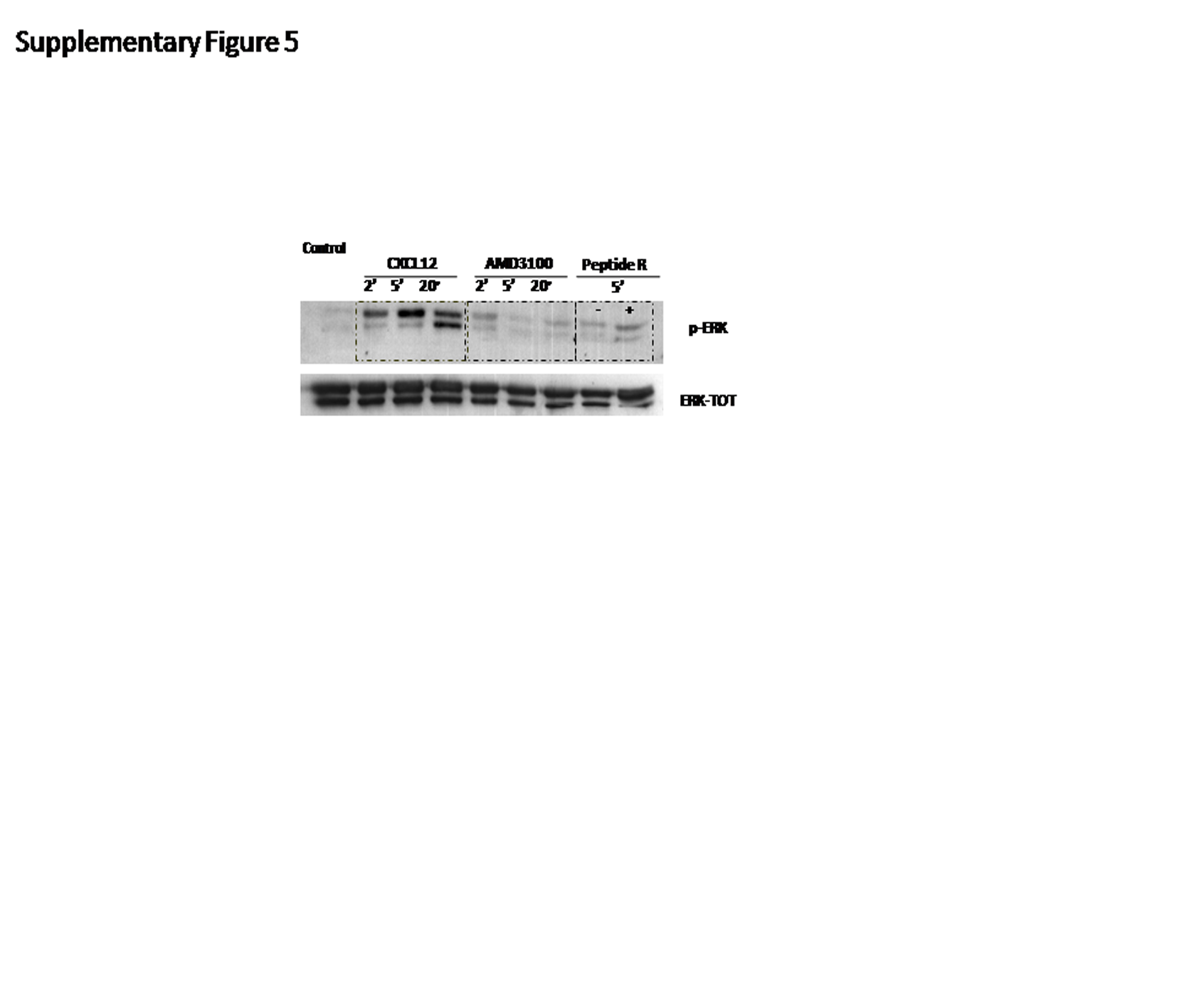

Supplement: Figure S5 — Peptide R inhibits the CXCL12 induced p-ERK. PES43 cell lines were serum starved for 16 hours. Then the cells were preincubated for 30 minutes with Peptide R (10 µM) or AMD3100 (10 µM) and then treated with CXCL12 (100 nM). (TIF) [file pone.0074548.s005.tif]
